# Practice, benefits, and impact of personal protective equipment (PPE) during COVID-19 pandemic: Envisioning the UN sustainable development goals (SDGs) through the lens of clean water sanitation, life below water, and life on land in Fiji

**DOI:** 10.1016/j.amsu.2021.102763

**Published:** 2021-08-26

**Authors:** Aneesh A. Chand, Prashant P. Lal, Kushal A. Prasad, Kabir A. Mamun

**Affiliations:** School of Information Technology, Engineering, Mathematics, and Physics (STEMP), The University of the South Pacific, Suva, Fiji

**Keywords:** COVID-19, Personal protective equipment (PPE), SDGs, Microplastic, Single-use-plastics, Pollution

## Abstract

**Background:**

The outbreak of coronavirus disease (COVID-19) highlights the global health emergency. To limit the rate of COVID-19 transmission to health care workers, adequate personal protective equipment (PPE) are required. Emerging reports indicate that the widespread usage of PPE during the COVID-19 outbreak has exacerbated plastic contamination in the ocean.

**Purpose:**

This paper attempts to understand the influence of practice, benefits, and impact of PPE during the COVID-19 crisis on clean water sanitation, life below water, and life on land (SDGs 6, 14, and 15 respectively) in Fiji and assess the effectiveness of measurements taken to deal with this crisis. Fiji is a small Pacific Island Country (PIC) and the global crisis of COVID-19 entered the Fijian border on 19th, March 2020. The second wave of COVID-19 was reported on 18th, April 2021, which began at a managed quarantine facility after contact between a couple returning from India to Fiji and a soldier. Since then the number of cases has been increasing daily and posing a risk to the public.

**Materials and method:**

A personal observation was made to collect the PPE pollution on the streets, near roads, car parks, markets, and towns.

**Results:**

Widespread PPE pollution was noticed, and the common PPE found on the Vesivesi road in Suva, Fiji were facemasks (61.36%) and hand gloves (38.64%), as it is mostly used by the public, police officers, municipal waste management, shopping malls workers, and medical care workers. Face shield littering was limited due to fewer users.

**Conclusions:**

In response to the COVID-19 pandemic, this study stresses a great concern on enabling SDGs 6, 14, and 15 and how the use of PPE during this period has impacted the natural environment. It is critical to remember that managing PPE waste during a contagious pandemic should be treated as an emergency and handled quickly.

## Highlights

• The single use of PPE has impacted the environmental sustainability.
• COVID-19 precautionary measures are impacting water resource.
• COVID-19 precautionary measures are affecting marine life and animals.
• To be sustainable, the policy-industry-research link must be strengthened.

## Graphical abstract

**Figure:**
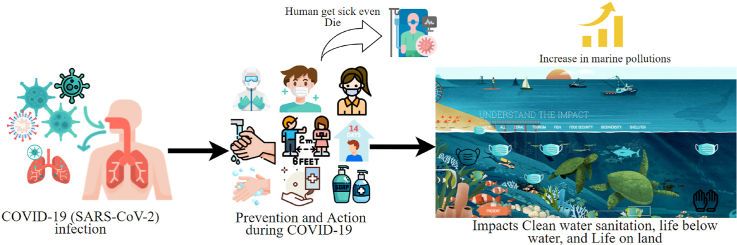

## 1 Introduction

**Fig. 1:**
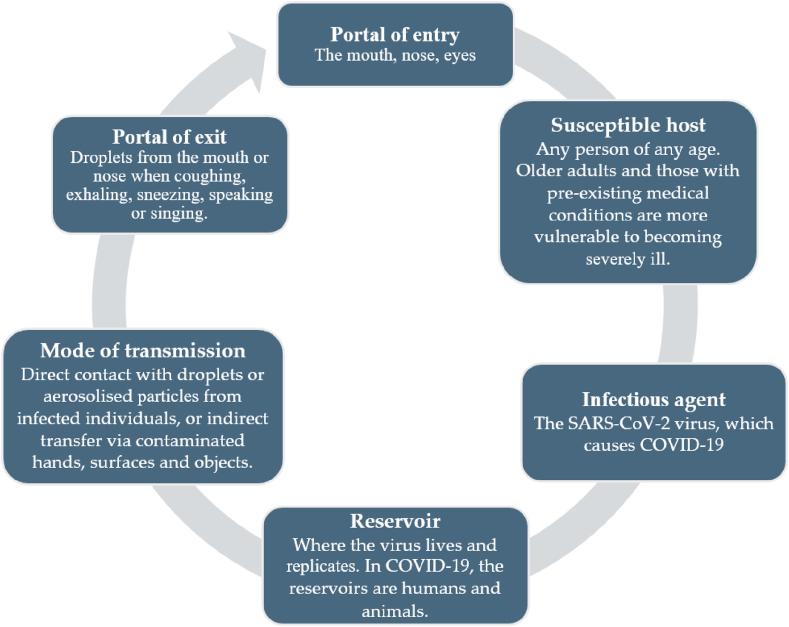
The COVID-19 infection chain.

The novel, threatening, and deadly coronavirus (COVID-19) crisis has potentially confirmed that the use of personal protective equipment (PPE) and health measures are very important [1,2]. By the end of 2019, a new deadly virus called SARS-CoV-2 was discovered in Wuhan City, Hubei Province, China [[3], [4], [5], [6]]. Currently, a significant amount of research has been conducted in reasoning the effect of this virus on social wellbeing [[7], [8], [9], [10], [11], [12]]. It is well known that this virus transmits from one person to another mainly through aerosolised respiratory droplets or particles, hence, to control the spread of this virus simple steps are given in Fig. 1 [13].

With the ever-increasing concern about this virus, the public followed all safety precautions which were directed by World Health Organization (WHO) [14,15]. The common method used to control the spread of the SARS-CoV-2 virus is through the rational use of PPE in health care and community settings, as well as during the handling of cargo. In this context, PPE includes gloves, surgical masks, goggles or a face shield, and gowns, as well as for specific procedures, respirators (i.e. N95 or FFP2 standard or equivalent) and aprons [[16], [17], [18]]. This reduces the risk of health workers transmitting the SARS-CoV-2 virus to others, or becoming infected with the virus themselves.

Furthermore, to prevent the transmission of the SARS-CoV-2 virus, health professionals are generally urged not to reuse their PPE, signifying that lots of plastic medical waste are generated daily. To restrict the community spread of COVID-19, the WHO has suggested the individuals to wear proper PPE (i.e., hand gloves, face masks, and face shields), social distancing, frequent handwashing, and limiting interpersonal interaction to outside situations, and closure of educational institutions in most countries [2,6]. Also, practically every country has advocated using facemasks to limit human-to-human transmission and protect the most sensitive and at-risk individuals. As a result, millions of facemasks have been created, used, and destroyed every day in accordance with this guideline and strict directives.

**Fig. 2:**
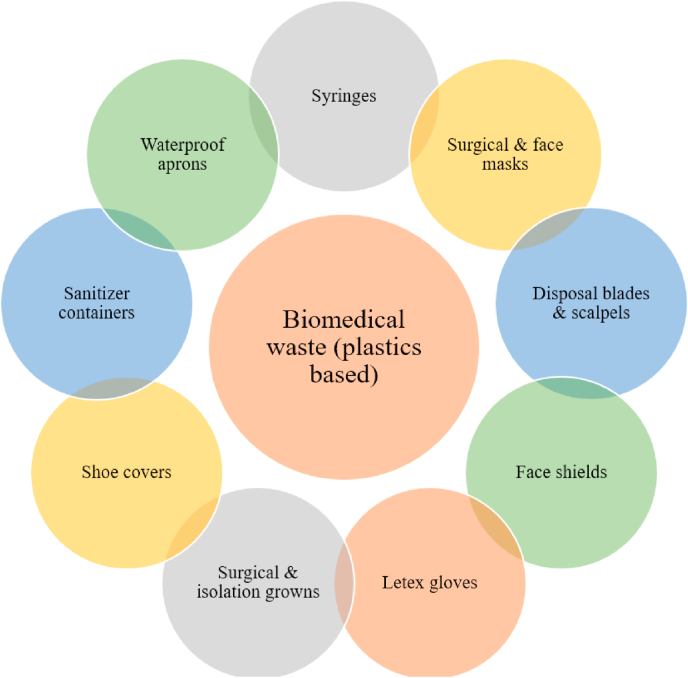
Types of plastic based biomedical wastes generated during the COVID-19 pandemic.

The majority of single-use PPE used by healthcare professionals and the public at large is thrown daily. The commonly used materials are given in Fig. 2. Apart from the health concerns raised by the COVID-19 crisis, this pandemic has exacerbated the problem of microplastics by causing an increase in consumer demand for single-use products and materials for safety purposes. The common places where medical related pollutions were seen are parking lots, medical facilities, beaches, roads, and shopping malls.

Notably, the footprint of COVID-19 and all the risk associated with it was experienced in small Pacific Island Countries such as Fiji [19,20]. Fiji is one of the developing states, consisting of over 300 small islands, and has a population of almost 902,004 as of May 11, 2021, based on Worldometer elaboration of the latest United Nations data [21]. The two major islands are Viti Levu and Vanua Levu. Fiji is a Pacific Island Country (PIC) and the spread of this virus was first reported on 19th, March 2020. Currently, the second wave of COVID-19 is affecting the Fijian citizens [20,22,23]. The widespread of this virus has led to many lockdowns and isolations.

To reduce the spread of COVID-19 in Fiji, it was enforced to use the proper PPE which is used to regulate the number of COVID-19 cases [1,2,9,13,16,17]. Health-care staff must take additional measures to protect themselves and avoid transmission in the workplace. PPE should be used properly by health care staff caring for COVID-19 patients, which includes choosing suitable PPE and being trained on how to put it on, its removal, and disposal [16,17]. On the other hand, the use of PPE has posed a greater risk to the environment in which suitable disposal process is not applied for used PPE. The ongoing COVID-19 pandemic has largely utilized single-use plastics globally, hence this has led to take-make-use-throw business. To cope up with the large production, use, and disposal of PPE, this event presented new challenges to traditional waste management systems. On the same note, improper disposal of PPE has raised a serious concern for the marine environment. In Fiji, it was evident that a huge number of PPE was found near sea, river, and picnic areas and this will be continued in coming years as daily usage of such materials is noticed [24]. Apart from the health concerns, this pandemic has contributed to Political, Economic, Social, Technological, Legal, and Environmental (PESTLE) issues. Section 2 gives an overview of the PESTLE analysis.

**Fig. 3:**
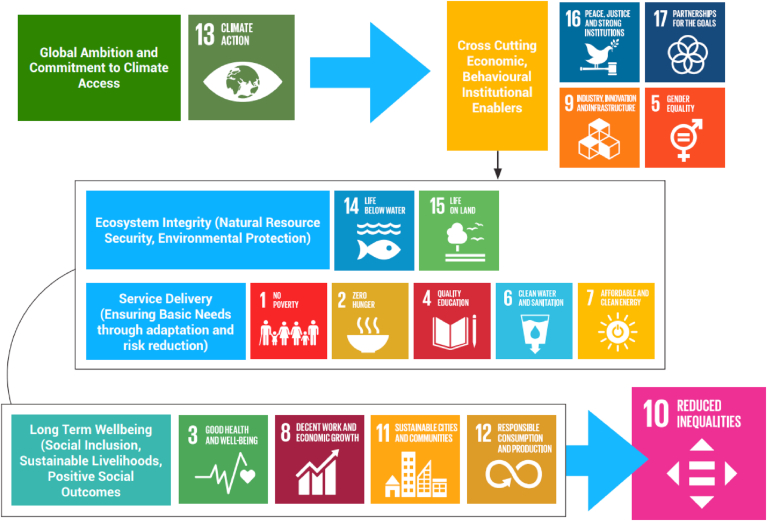
Depicts the ACT which Fiji government has put in place with National Development Plan (NDP) and UN SDGs.

In 2017, the Fijian Government launched its 5-Year & 20-Year National Development Plan (NDP) with the vision of transforming Fiji with sustainability and combat climate change as given in Fig. 3 [25]. In addition, the 2030 Agenda, when combined with the Paris Agreement, is revolutionary and has a high development objective. Amongst the 17 SDGs, clean water sanitation, life below water, and life on land are SDGs, 6, 14, and 15, respectively, and they set a platform to combat the issue raised with COVID-19 (i.e. environmental pollution) [25]. On the same note, the geographical location of Fiji is such, where access to ocean and river is quite oblivious and due to improper disposal of PPE, a serious concern is raised. The past and current wave of COVID-19 has made noticeable environmental pollution around beaches, seabed, near roads, bus stations, car parks, markets, towns, and cities, as given in Fig. 3. All these finally end up in the ocean creating a serious problem with marine life, due to micro-plastic present in PPEs.

Finally, the act which is placed by the Fijian government needs to be given much attention in the following areas; combat for climate change and promote sustainability. Therefore, this research layouts and suggests the need of PPE, disposal methods, and its impact on the natural environment. This research is to fill the current knowledge gaps regarding COVID-19 and the impacts associated with PPE pollution and lay groundworks for better waste management systems in Fiji.

## 2 Background

Studies have revealed that the most serious drawback of the COVID-19 pandemic is the negligence on environmental issues, which has resulted in global crises due to higher micro-plastic pollution. The WHO has confirmed that standard PPE are single-use, and after use, it becomes a harmful medical waste [16,17]. Yu et al. [26], clarified that COVID-19 has generated substantial amounts of harmful waste globally while Feng et al. [27], showed that China, Japan, South Korea, and other countries have given instructions to wear masks in public. Considering this, there is an immeasurable singleuse of plastic materials, so it is essential to take measures in preventing the spread of the infection by the use of proper PPE and its appropriate disposal method. Due to the state of emergency, the general public, medical officers, police, and security officers are required to use PPE, particularly face masks, gloves, face shields, and use proper disposing mechanisms. Also, a lot of environmental issues has been raised during this period, i.e., the used PPE are found stranded on the beaches, coastlines, rivers, and are littering cities which directly impact three different SDGs. Fig. 3 illustrates the implication of PPE on clean water sanitation (SDG6), life below water (SDG14), and Life on land (SDG15).

**Fig. 4:**
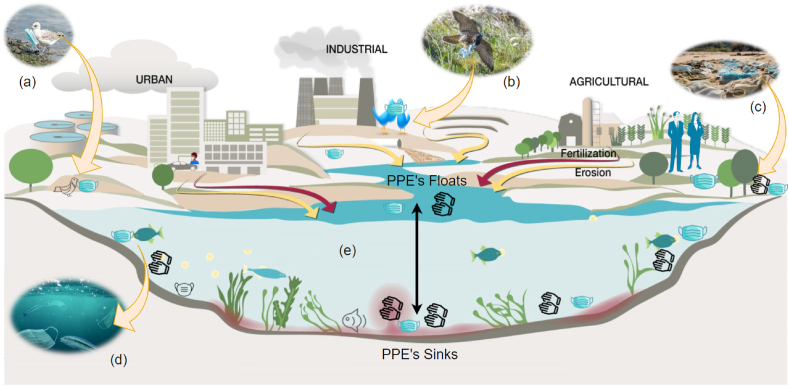
A graphical representation of PPE pollution caused due to COVID-19.

Fig. 4 depicts, land pollution, air-pollution, and water pollution. Fig. 4 (a) and (b) conveys that the improperly disposed PPE materials are picked by birds or animals which can act as a carrier and the spread of virus to humans. Also, it can be a medium to transfer used PPE into the water streams and seas. Fig. 4 (c) shows human negligence on the disposal of the PPE used by the medical staff, general public, business communities, and waste management departments which can lead to a polluted environment and a hindrance to health safety. Fig. 4 (d) and (e) shows a sad reality that the disposable PPE end up in our marine ecosystem posing danger to the marine lives and an unsafe water body. Nonwoven materials (e.g., spunbond and meltblown spunbond) are used to make most disposable face masks, with polypropylene and polyethylene. Hand gloves are generally made of plastic materials with low-density polyethylene, nitrile, latex, and vinyl, mostly it has high chemical and mechanical resistance, resulting in high persistence in the atmosphere following dispersion. Hence, it is clear that PPE poses a lot of environmental challenges when it is not properly disposed.

The above representation reveals that poor PPE waste management in the COVID-19 pandemic has significantly impacted marine life, freshwater, and life on land. Table 1 summaries the steps involved in handling the PEE-. According to Fadare and Okoffo, some PEE- have composite materials with synthetic non-degradable polymers which makes them either float or sink depending on their characteristics [28]. De-la-Torre et al. [29], shared an observation on types of plastic pollutants. It was evident that some PPE can persist in the ecosystem for extended periods, being equally susceptible to surface open ocean, while others may appear submerged in the sediments, eventually becoming part of the geological record. Studies have indicated that seawater has a massive amount of micro-plastic [30]. Apart from other marines, micro-plastic pollution during COVID-19 pandemic has greatly impacted the clean water sources, marine life, and animals.

**Table 1:** Different steps involved in handling the PPE.

Type	Protection	Appropriate for	Use guidelines	Reuse	Fabrication
Homemade (cloth mask)	Huge droplets from coughs or sneezes can be protected.	Members of the society who abide by the laws.	People who wear must maintain physical distance, wash their hands often, and avoid touching their ears.	If properly washed, it can be used again.	It is cheap and easy to make with breathable fabrics like cotton or cotton blends.
Clinical mask	Fluid-resistant and capable of filtering small particles.	For front-line health care providers	Jobs in the medical field must adhere to the rules of the organization.	Health workers should follow institutional protocol.	Fabrication standards must be followed when using medical-grade polypropylene.
N95 Respirator	Filters 95% of very small particles when tightly fitted.	For front-line health care providers. More masks are required for healthcare professionals, so community use is discouraged.	To be completely accurate, skilled fit-testing is needed.	Following CDC guidelines for long-term use or reuse is needed.	Specialized materials and processes were used to create this product.

### 2.1 Overview of COVID-19 pandemic in Fiji

**Fig. 5:**
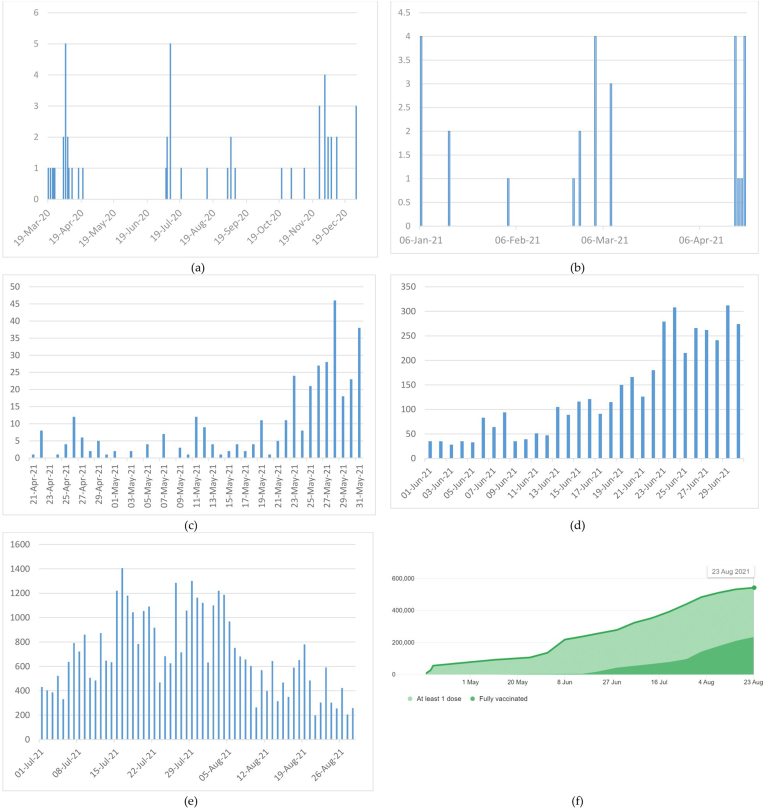
Fig. 5 illustrate the number of COVID-19 cases the country has encountered per day (a) shows the COVID case reported in 2020, (b) shows before the second wave cases, (c), (d), (e) are the trend of COVID-19 cases for second wave (as of 28 August), and (f) is vaccination for 1st nd 2nd doses (as of 23 August,2021) [31].

The first case of COVID-19 was reported on the 19th of March 2020 [20]. Alongside this, between March 20th and April 18th, a total of 54 new cases were confirmed of which, 37 cases either were related to international travel or epidemiologically linked to international travel, while the remaining 18 cases were thought to be spread across the population, as givn in Fig. 5(a). Throughout this phase, 53 people (cases) recovered, but there were two deaths in the country due to COVID-19 [20]. The happiest period for Fiji was from 19th April to 5th July when there was no positive case recorded. The cases which occurred after 6th July were associated with international travel while undergoing a mandatory 14 days quarantine period in Nadi at the international border quarantine facilities. This was considered the first wave of COVID-19 for the country.

**Fig. 6:**
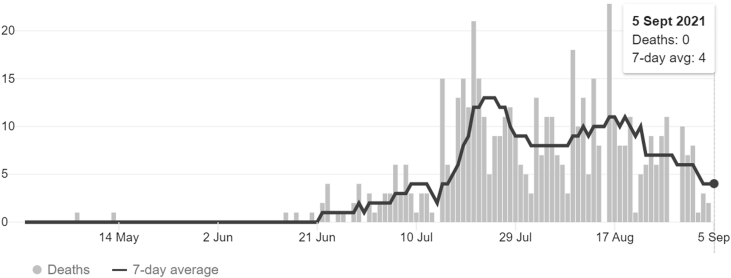
Number of deaths caused by COVID-19 in Fiji.

Sadly, due to a breach at the international quarantine facility, Fiji was hit by the second wave of the COVID-19 pandemic. Now, the country again has locally transmitted cases, border quarantine cases as well as community transmitted cases. As of 6th September, the country has 19,463 active cases. Looking at the current (April,2021) outbreak a total of 47,923 cases has been reported and overall 47,993 cases since the first outbreak which started in March 2020 [23]. On the bright side, a total of 32,728 individuals have recovered from COVID-19. Unfortunately, there has been 508 death cases reported during COVID-19 pandemic in Fiji of which 506 deaths are from current outbreak and remaining 2 deaths from previous outbreak., as given in Fig. 6. In addition, a total of 340,217 samples have been tested since this outbreak started in April 2021, with 383,078 tested since testing began in March 2020. On 5th September a total of 774 tests have been reported [31]. Also, the country is fast moving on vaccinating the public to go back to its normal. As of 03rd September, the total number of people received 1st and 2nd dose of COVID-19 vaccine are 566,210 and 299,943 respectively [31], as given in Fig. 5 (f).

Fig. 5 illustrates the number of COVID-19 cases the country has encountered per day. Fig. 5 (a) shows the COVID-19 cases reported in 2020 while Fig. 5 (b) shows before the second wave cases. The increasing trend of COVID-19 cases are shown in Fig. 5 (c), (d), and (e). The second wave of the virus is relatively much higher compared to the first wave in 2020. One of the main contributing factor for the increasing number of cases is negligence of COVID-19 protocols. The Delta Variant known as B.1.617.2 which was discovered in India, has also been found in the country following recent testing [31]. This has caused a lot of fear in citizens as well as the thought of lockdown. Due to such reasons people have disregarded the COVID-19 protocols such as social distancing, use of face masks, sanitizers, etc.

### 2.2 Categories of PPE used in Fiji

**Fig. 7:**
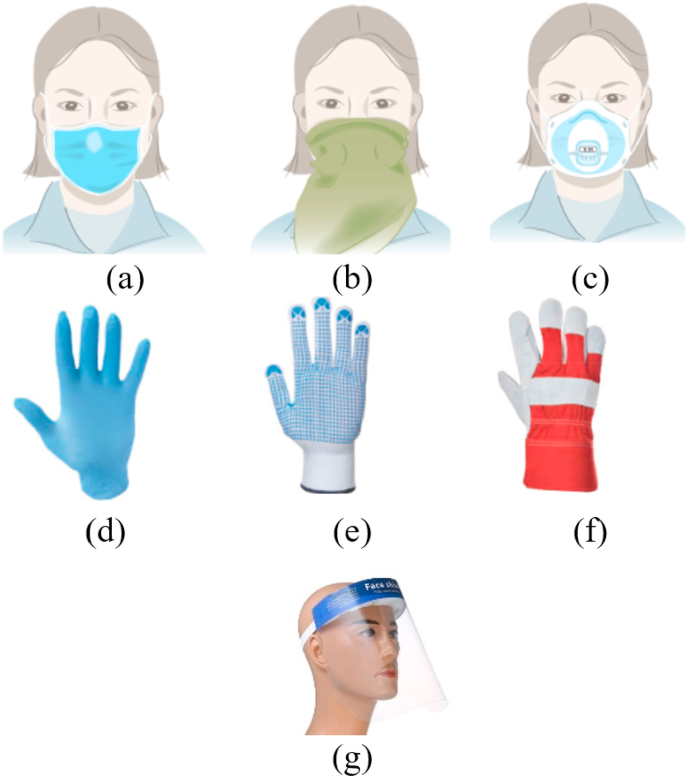
Different PPE used in Fiji.

Facemasks and hand gloves are two of the most widely worn PPE in Fiji, but face shields are still used in some convenience stores. Public wear face mask when they move out, commonly homemade cloth masks are used. The common type of PPE used and the materials utilized in its production are given in Fig. 7 and Table 2.

**Table 2:** Different types of PPE used in Fiji during the COVID-19 pandemic.

Common PPE	Different types of the PPE	Fig. Reference
Facemasks	*Surgical mask* Material used – non-woven fabric/polypropylene Benefits – One way protection. Captures particles or droplets from wearer only.	Fig. 7(a)
	*Homemade (DIY)* Material used – cotton, chiffon, and silk. Benefits – Could help stem rocketing infection rates	Fig. 7(b)
	*N95* Material used – synthetic plastic fibers usually polypropylene. Benefits – Two way protection. Filters air entering/exiting the wearer. – Filters at least 95% of airborne particles.	Fig. 7(c)
Hand gloves	*Disposable gloves* Material used – nitrile, latex, or vinyl Benefits – protection against mild irritants.	Fig. 7(d)
	*Fabric gloves* Material used – cotton or fabric Benefits – insulate from heat and cold. – Enhance grip for better handling.	Fig. 7(e)
	*Leather gloves* Material used – leather Benefits – protection from rough abrasive surfaces.	Fig. 7(f)
Face shields	Material used – polycarbonate and polyester Benefits – Protects eyes. – Prevent individuals from touching their faces constantly. – Complements masks.	Fig. 7(g)

In general, to overcome the serious issue of marine pollution caused by inappropriate PPE disposal, it is suggested that, low-density polymers should be used as artificial substrates for rafting non-native or invasive organisms. Another great and important research could provide a better solution if organic materials can be used for face masks.

## 3 Practice, benefits, and impact of PPE during the COVID-19 pandemic

**Fig. 8:**
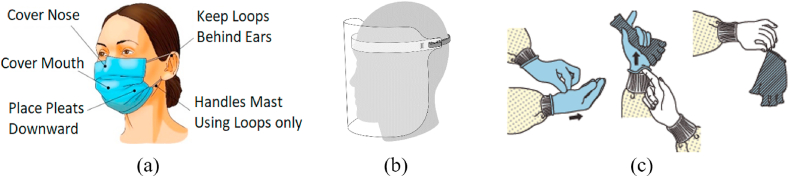
Practice of PPE during the COVID-19 pandemic.

Earlier it has been mentioned that facemasks, hand gloves, and face shields are amongst the most popular PPE used in Fiji. The major benefit of wearing PPE is that it reduces the risk of spreading SARS-CoV-2 virus. This will be achieved when the transfer rate will decrease, because the virus will be contained within the PPE. Thus, there will be a lower risk posed to health and safety of surrounding individuals [32]. Fig. 8 demonstrates the proper way of wearing these PPE [17]. The impacts caused by improper disposal of PPE are given in Table 3.

**Table 3:** Impacts of improper disposal of PPE in Fiji during the COVID-19 pandemic.

	Impacts
Human	Improper disposal of PPE gives additional work to municipal waste management staffs. It also improvises their health and safety as they get more vulnerable.
Environment	Land and water pollution. Due to decomposition and weathering, the plastic material gets washed into the ocean which affects under water ecological life.
Animal	PPE lying on land tends to be a play materials for dogs, birds, and cats. As a result they become carriers of SARS-CoV-2 virus and in return affect humans.

## 4 Results and observation

**Fig. 9:**
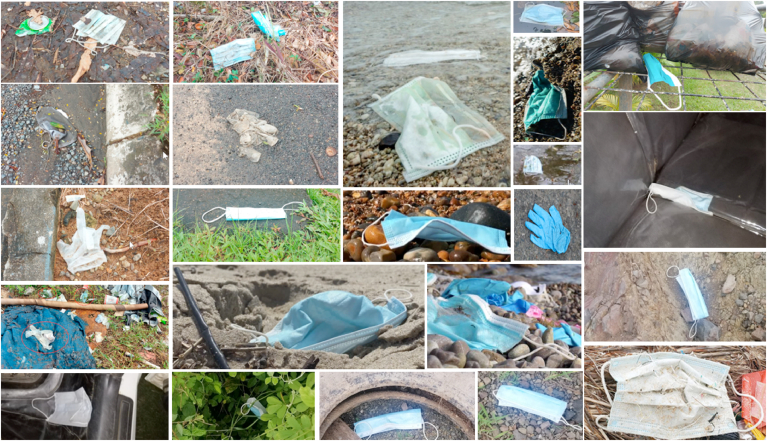
Different PPE pollution found in Fiji (personal collection).

It is evident that the COVID-19 pandemic has increased pollution which will pose a great threat and environmental challenges in coming years. In surveys conducted by the researchers, PPE were found in coastal areas, near roads, bus stations, car parks, markets, towns, and cities, given in Fig. 9. This new variety of PPE pollution poses a challenge to top predators in the ocean, as well as providing a source of microplastics.

**Fig. 10:**
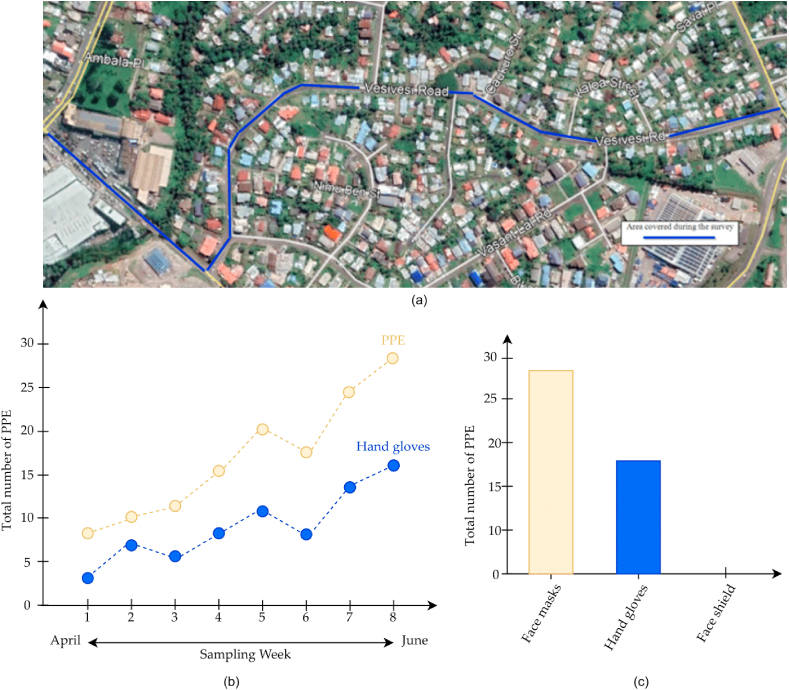
(a) Map of the region and sampling site, (b) cumulative number of PPE across sampling site, and (c) total number of face masks and hand gloves.

The occurrence of PPE was surveyed during the COVID-19 pandemic, data collected from 23rd April to 11th June 2021. Fig. 10(a) shows the area which is surveyed in Suva, Fiji. During the eight weeks of samplings, and eye-catching survey discovered a total of 44 items across sites. Face masks was the most frequent item found throughout the survey period, accounting for 27 of the total (61.36%), followed by 17 hand gloves (38.64%), as shown in Fig. 10(c). Interestingly, no face shields were found on any site. Even though these monitoring durations may not be sufficient to conclude that PPE pollution is expanding over time, but it is important to note that there will be a growth in the coming months as COVID-19 cases are increasing.

These values were used to calculate the PPE density in each sampling site as described by Ref. [33].

(1) $$C=\frac{n}{a}$$

where, *C* is the density of PPE per m^2^,*n* is the number of PPE counted, and *a* is the surveyed area.

Due to negligence of people, lack of environmental awareness, and poor municipal waste management practices are the root causes of this issue. Only a few articles have reported PPE pollution in coastal environments in Fiji Islands. Hence, it is important to put effort into PPE pollution research to have a better understanding of the impact all across the environmental compartments.

## 5 Discussion

At first level, the COVID-19 pandemic seems to be implicitly leading to the UN 2030 SDGs (namely 6, 14, and 15 SGDs) by increasing the risk towards clean water sanitation, life below water, and life on land. It is a sad reality that the PPE utilized in the due course of the COVID-19 pandemic are becoming a health and environmental concern. It is very important to advise and educate the public on the impacts of inappropriate PPE disposal on the health and environment. It is suggested to address this unethical practice at an early stage before the problem starts replicating and becomes irreversible.

### 5.1 SDGs vs. COVID-19 impact

One of the most significant SDG goals is to combat climate change; consequently, SDGs 6, 14, and 15 are studied in this research, as well as how COVID-19 has had a thought-provoking impact towards achieving these goals and are given in Table 4.

**Table 4:** Impact and Drawback of PPEs pollution on SDGs.

Goals	End life of PPEs	Drawback
SDG 6: Clean Water Sanitation	Used PPE are not properly disposed and thrown near beaches, seabed, near roads, bus stations, car parks, markets, towns, and cities	Normally it ends up in rivers, sea, and small creeks. Hence, it contaminates rivers and fresh water which may be used for washing or drinking purpose. Also, animals consumes river water for drinking and breeding, but presence of micro particles causes many issues. Different water colour is observed once the PPE end up in rivers.
SDG 14: Life Below Water		The small size of micro plastics results in their uptake by a wide range of aquatic species disturbing their physiological functions, which then go through the food web creating adverse health issues in humans.
SDG 15: Life on Land		Used PPE lying on ground causes environmental pollution. Visually it is not appealing. It also can transmit the virus to the other person who does the cleaning or by mistake steps on it with open sores. Animals such as birds, cats, and dogs are normally attracted to wastage materials. They can also become sick if it is eaten by mistake (when mixed with other waste product).

### 5.2 PESTLE analysis of COVID-19 in Fiji

**Fig. 11:**
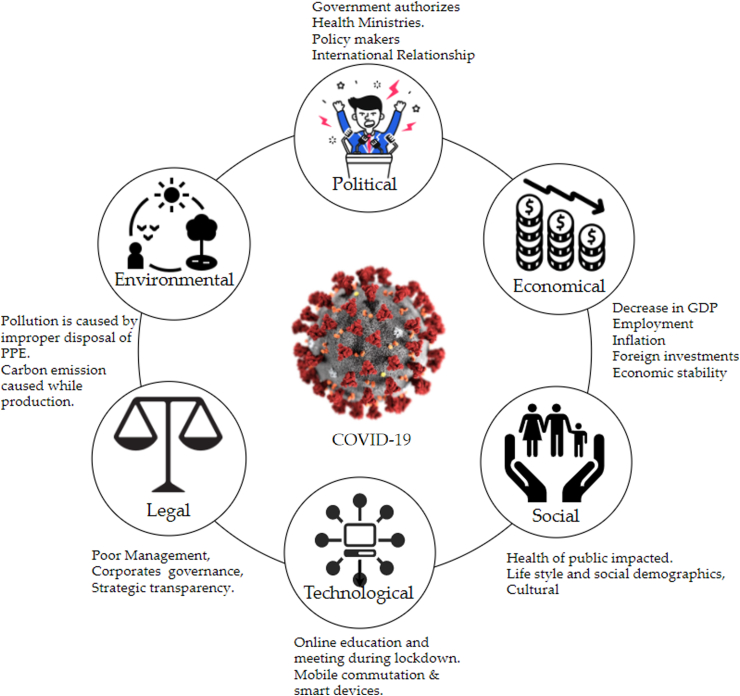
Systematic representation of PESTLE for COVID-19.

The COVID-19 pandemic can be further classified into six categories and each can be differently studied. It is important to comprehend the PESTLE analysis. The *Political*, *Economical*, *Social*, *Technological*, *Legal* and *Environmental* (PESTLE) are the popular pillars which are explained in below subsection. A graphical representation of PESTLE is given in Fig. 11.

***Political Concern*:** Government authorities' role is critical as they set laws and orders during this time. Health Ministries are obliged to provide proper health facilities. At this stage, controlling people's movement is a great concern as a lot of effort is taken to achieve less local movement. International relationship is also a concern, as assistance is provided in terms of vaccines, foods, and capital.

***Economical Concern:*** The pandemic has tremendously impacted world gross domestic product (GDP), particularly for the developing island countries. Currently the hotcake discussion globally is inflation, economic stability, unemployment, and local & foreign investments.

***Social Concern:*** The rapid transmission of SARS-CoV-2 virus has a great impact across countries in many ways including; lifestyles, business & social cultural structure, demographics, and people's lives and communities.

***Technological Concern:*** During the lockdown, smart devices such as mobile communication, internet and bio-machines offered a big advantage. Globally the Internet of Things (IoT) smart devices and technological advances have promoted online education, work from home, and conducting virtual international meeting.

***Legal Concern:*** Globally different legal agendas have largely impacted countries due to their set of rules for social behaviour. Poor management of peoples’ movement has been related to corporate governance, disclosure, contracts, financing, strategic transparency, employment and others.

***Environmental Concern:*** Looking at the current situation, this virus has taken lives of millions of innocent individuals in many countries across the world however on the flip side, nature has shown some of the positive responses due to lesser movement thus lesser pollution (lower carbon emission). But COVID-19 created a serious concern in regards to land and water pollution due to improper disposal of face mask and hand gloves. The drawback of PPE pollution with impacted SDGs is summarized in Table 4.

### 5.3 Disposal method

**Fig. 12:**
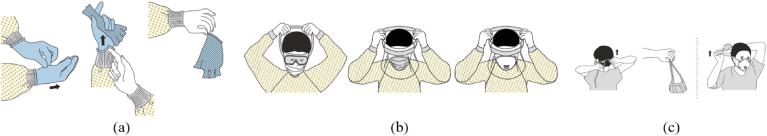
Different types of PPE disposal method.

Prominently, improper PPE disposal causes a lot of threats to human, animal, and environment. One of the significant processe involved which needs to be followed during COVID-19 is the disposal of PPE correctly. Fig. 12 shows some of the processes associated with the PPE disposal. Table 5 gives generalized disposal steps for each of the common PPE used in Fiji. It is important to take note and follow each step in the right way so that the local transmission of SARS-CoV-2 virus is controlled. Having said that, wearing PPE does not ensure 100% protection, but it can definitely reduce the transmission of this deadly virus.

**Table 5:** Disposal Steps of various PPE.

PPE Type	Disposal description	Figure
Hand gloves	As the gloves may be contaminated, it is advisable to remove the gloves using an appropriate procedure. The procedure is to remove one glove by grasping the palm area of the other and hold it in the gloved hand. Remove the other one by sliding fingers from the wrist end peeling it off and disposing it properly. If the hand is contaminated while removal, it is recommended to wash your hands or sanitize it.	Fig. 11(a)
Face shield	The face shield may be contaminated so it is advisable to remove by unstrapping the head band or removing the ear bands. Dispose it properly. If the hand is contaminated while removal, it is recommended to wash your hands or sanitize it.	Fig. 11(b)
Face masks & respirator	The mask may be contaminated therefore do not touch it. Remove the mask without touching the front but the ear straps around the ear. Dispose it correctly in a disposal container. If the hand is contaminated while removal, it is recommended to wash your hands or sanitize it.	Fig. 11(c)

In addition, encouraging the use of reusable face masks is an important strategy to reduce PPE pollution. Considering that one of the major sources of plastic pollution in Fiji is a lack of environmental awareness, long-term programs are needed to change citizens' attitudes and encourage sustainable practices that can help prevent future plastic pollution.

Consequently, different governing authorities have put in place some effective waste management measures during the pandemic. For a sustainable waste disposal exercise during the COVID-19 pandemic Table 5 summarizes the procedures.

## 6 Conclusion

Like other countries, Fiji is also experiencing the impact of COVID-19 and it is critical to report its threatening impact in literature. In this research, the effect of high usage rate and incorrect disposal of PPE during COVID-19 has shown a huge environmental impact. Globally, the outcomes are similar, improper PPE disposal practices are a major source of concern for human and environmental health. In Fiji, due to negligence and poor incineration facilities, used PPE from residential areas causes threatening challenge for municipal waste management and is a leading carrier for aquatic ecosystem pollution. Hence, due to recurrent outbreaks of COVID-19, extensive use of PPE by the public is critical to avoiding the pandemic's extreme negative environmental consequences. This research was conducted during the lockdown period and the following observation were made.

• Littering of facemasks was more frequent compared to hand gloves, 61.36% was accounting for face masks and followed by 38.64% for hand gloves. Mostly public uses face masks, hand gloves are used by medical practitioners, police officer, municipal waste management, and shopping malls workers in Fiji.
• There are limited face shield litters as it is commonly used in shopping malls, hospitals and restaurants.

Due to the negligence caused by Fijian citizens, a huge number of used PPE are ending up in marine ecosystem which is a serious and alarming concern. It is important to prevent such activities by following the simple steps involved in PPE disposals. A PESTLE analysis is discussed to weight the challenges which is associated with COVID-19. Use of PPE during this COVID-19 crisis is valid as the spread of the virus can be contained, however if the correct disposal of used PPE is not practiced then the use of PPE for such situation is illogical and irrational. The following recommendation will surely improve the negative impact of PPE.

• Create awareness (educate) on the impact caused by PPE pollution,
• Warning on packaging,
• Standardize government waste policies,
• Policies to set on biodegradable masks,
• Penalise if found littering, and
• Use unmanned aerial vehicles (UAV) for monitoring of PPE pollution,

## Provenance and peer review

Not commissioned, externally peer-reviewed.

## Funding

No funding received.

## Ethical approval

Not applicable – No IRB review required.

Not a human study.

## Research registration Unique Identifying number (UIN)

1. Name of the registry: N/A
2. Unique Identifying number or registration ID: N/A
3. Hyperlink to your specific registration (must be publicly accessible and will be checked): N/A

## Author contribution

Aneesh A. Chand was the lead author on this letter.

Aneesh A. Chand – Concept development, study design; data collection, writing original draft, editing drafts, approval of final article.

Prashant P. Lal and Kushal A. Prasad– Concept development, data analysis, resources, writing and editing of manuscript.

Kabir A. Mamun - Review of manuscript, writing, and editing.

## Guarantor

Aneesh A. Chand.

## Declaration of competing interest

No conflicts of interest.
